# Cardiovascular risk management among individuals with type 2 diabetes and severe mental illness: a cohort study

**DOI:** 10.1007/s00125-024-06111-w

**Published:** 2024-02-26

**Authors:** Jonne G. ter Braake, Kelly J. Fleetwood, Rimke C. Vos, Luke Blackbourn, Stuart J. McGurnaghan, Sarah H. Wild, Caroline A. Jackson, on behalf of the Scottish Diabetes Research Network Epidemiology Group

**Affiliations:** 1 Department of Public Health and Primary Care, Leiden University Medical Centre, The Hague, the Netherlands; 2 Usher Institute, University of Edinburgh, Edinburgh, UK; 3 MRC Institute of Genetics and Cancer, University of Edinburgh, Edinburgh, UK

**Keywords:** Bipolar disorder, Cardiovascular risk, Clinical care, Cohort study, Depression, Diabetes management, Health disparities, Mental disorders, Schizophrenia

## Abstract

**Aims/hypothesis:**

The aim of this study was to compare cardiovascular risk management among people with type 2 diabetes according to severe mental illness (SMI) status.

**Methods:**

We used linked electronic data to perform a retrospective cohort study of adults diagnosed with type 2 diabetes in Scotland between 2004 and 2020, ascertaining their history of SMI from hospital admission records. We compared total cholesterol, systolic BP and HbA_1c_ target level achievement 1 year after diabetes diagnosis, and receipt of a statin prescription at diagnosis and 1 year thereafter, by SMI status using logistic regression, adjusting for sociodemographic factors and clinical history.

**Results:**

We included 291,644 individuals with type 2 diabetes, of whom 1.0% had schizophrenia, 0.5% had bipolar disorder and 3.3% had major depression. People with SMI were less likely to achieve cholesterol targets, although this difference did not reach statistical significance for all disorders. However, people with SMI were more likely to achieve systolic BP targets compared to those without SMI, with effect estimates being largest for schizophrenia (men: adjusted OR 1.72; 95% CI 1.49, 1.98; women: OR 1.64; 95% CI 1.38, 1.96). HbA_1c_ target achievement differed by SMI disorder and sex. Among people without previous CVD, statin prescribing was similar or better in those with vs those without SMI at diabetes diagnosis and 1 year later. In people with prior CVD, SMI was associated with lower odds of statin prescribing at diabetes diagnosis (schizophrenia: OR 0.54; 95% CI 0.43, 0.68, bipolar disorder: OR 0.75; 95% CI 0.56, 1.01, major depression: OR 0.92; 95% CI 0.83, 1.01), with this difference generally persisting 1 year later.

**Conclusions/interpretation:**

We found disparities in cholesterol target achievement and statin prescribing by SMI status. This reinforces the importance of clinical review of statin prescribing for secondary prevention of CVD, particularly among people with SMI.

**Graphical Abstract:**

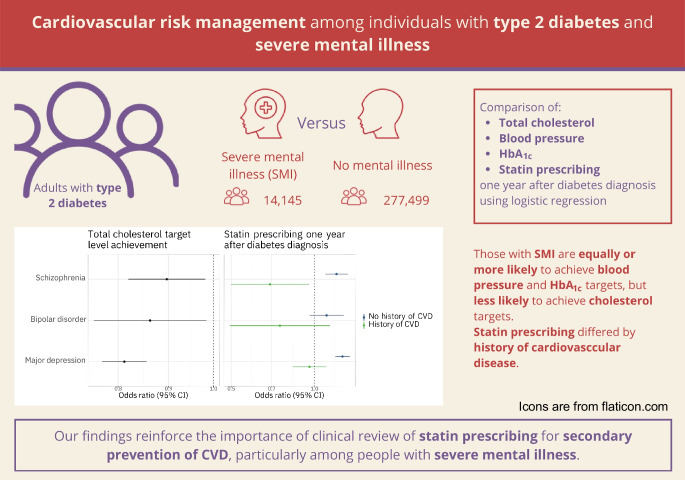

**Supplementary Information:**

The online version of this article (10.1007/s00125-024-06111-w) contains peer-reviewed but unedited supplementary material.


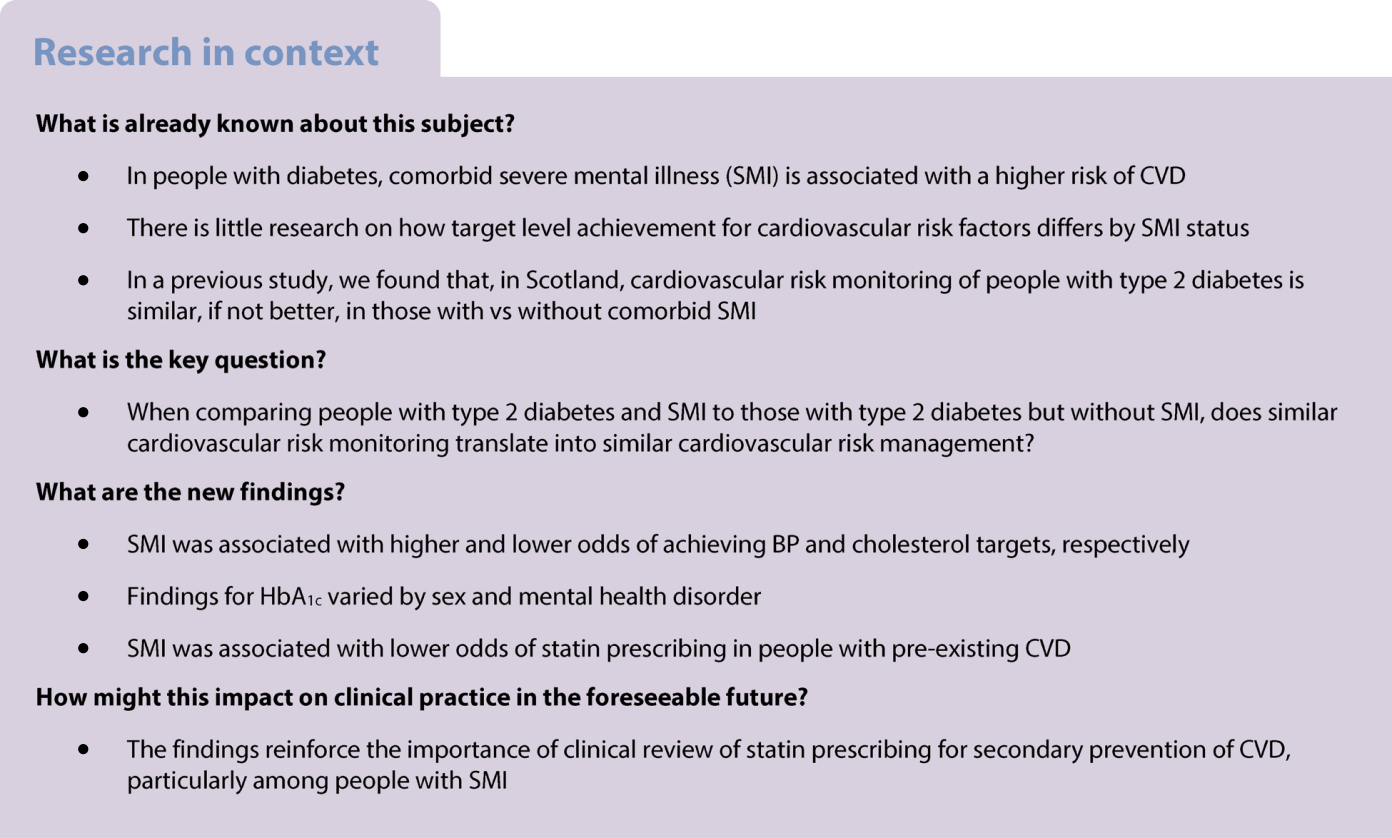


## Introduction

People with severe mental illness (SMI), defined here as schizophrenia, bipolar disorder or major depression, have a life expectancy that is reduced by 10–20 years compared with the general population [1–6]. This reduced life expectancy is largely due to natural causes, especially CVD [1–5, 7], for which diabetes is a major risk factor. Among people with type 2 diabetes, comorbid SMI is associated with poorer outcomes, including an elevated risk of micro- and macrovascular complications and increased mortality risk [8–10].

Mental health disparities in CVD-related diabetes outcomes are often assumed to be at least partly due to sub-optimal receipt of cardiovascular risk monitoring in people with SMI [11, 12]. However, despite receipt of routine diabetes monitoring being similar, if not better, in people with vs without SMI in Scotland [11], the former still have a higher risk of CVD-related outcomes [10]. These poorer outcomes may therefore be due to differences in the translation of cardiovascular risk monitoring into adequate risk management by SMI status.

Previous studies on SMI and cardiovascular risk management in people with diabetes have focused on investigating lipid levels [12–18], BP [13–15] and/or glycaemic levels [12, 14–18], as these key cardiovascular risk factors can be managed pharmacologically if lifestyle modification is ineffective [13–19]. The findings are contradictory, with some studies reporting no difference in risk factor levels [13, 14, 16] and others finding either better [17] or worse [12] lipid levels, better BP [15], and better [12] or worse [15, 18] glycaemic management among those with SMI. In the UK, clinical guidelines indicate that lipid-modifying medication (statins) should be offered to all those with diabetes over 40 years of age, irrespective of cholesterol levels [20]. Only two studies have compared prescribing of lipid-modifying medication by SMI status, reporting that compared to patients without mental illness, patients with SMI were less likely to receive a lipid-modifying medication prescription [13, 16]. However, the findings from existing studies are limited by either a small sample size [13], possible selection bias [14, 16–18] and conflicting findings [12–18]. Moreover, most studies did not differentiate between types of SMI [13–16, 18]. We sought to address these limitations by comparing achievement of lipid, BP and glycaemic targets 1 year after type 2 diabetes diagnosis by SMI status in Scotland, using a national cohort study. In secondary analyses, we compared prescribing of statins by SMI status.

## Methods

This paper is written in accordance with the STrengthening the Reporting of OBservational studies in Epidemiology (STROBE) and REporting of studies Conducted using Observational Routinely collected Data (RECORD) statements.

### Study population and design

**Fig. 1:**
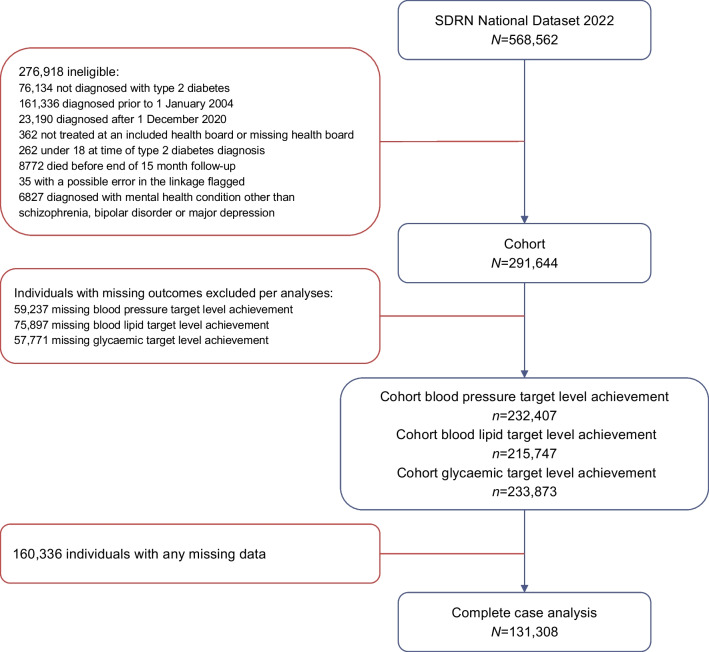
Flowchart showing selection of the study population. The analyses for achievement of BP, lipid and glycaemic target levels each have a unique cohort, as individuals with missing outcome data are excluded per outcome after multiple imputation (as described in ESM Methods). SDRN, Scottish Diabetes Research Network

We performed a retrospective cohort study using data from the Scottish Diabetes Research Network National Diabetes Dataset [21]. This dataset contains information on diabetes-related care from primary and secondary care settings for >99% of those diagnosed with diabetes in Scotland since 2004 (based on cross-validation of diagnoses in primary care records against hospital admission and prescribing records by the Public Health Scotland data linkage team), linked to various routinely collected health datasets [21]. We included all adults with a primary or secondary care record of a type 2 diabetes diagnosis between 2004 and 2020, who lived in a Scottish health board region. Individuals who died within 15 months after diabetes diagnosis, that could therefore not be followed up for 15 months, were excluded (Fig. 1).

### Definition of severe mental illness

We identified history of a mental health condition from diagnoses recorded in acute or psychiatric hospital admission records, available from 1981 onwards, using ICD-9 and ICD-10 codes (http://www.icd9data.com/2007/Volume1/default.htm and http://apps.who.int/classifications/icd10/browse/2016/en) (see electronic supplementary material [ESM] Table 1). We included mental health conditions recorded after individuals turned 18 years of age and before being diagnosed with type 2 diabetes. The mental illness groups were mutually exclusive. We used a severity hierarchy for individuals diagnosed with multiple mental health conditions, with schizophrenia as the most severe disorder, then bipolar disorder and lastly major depression [10]. We excluded individuals who only had a hospital admission record of any mental illness other than schizophrenia, bipolar disorder or major depression. We used the term major depression to align with that in previous studies, including on SMI and receipt of diabetes care in Scotland [10]. As in this previous study, we used a hospital record of depression as a proxy for severe depression, which we refer to as major depression [10].

### Outcomes

The primary outcome measures were serum cholesterol, BP and HbA_1c_ target level achievement by SMI status approximately 1 year after type 2 diabetes diagnosis. We included a 1-year endpoint to facilitate detailed investigation of target level achievement in the short term, given the paucity of SMI disorder-specific findings in the published literature and findings from a previous study suggesting that changes in cardiovascular risk factor levels occur largely within the first year after diabetes diagnosis [18]. We used the definitions used in the Scottish Diabetes Survey, an annual audit of quality of diabetes care, to define target levels as follows: total cholesterol ≤5.0 mmol/l; systolic blood pressure (SBP) ≤140 mmHg; HbA_1c_ <58 mmol/mol (7.5%) [22]. We used values for risk factors reported within 9–18 months after diabetes diagnosis, reflecting the flexibility in timing of the annual measurements in clinical guidelines. We calculated the median value when multiple measures were recorded during this period.

Our secondary outcome measure was prescription of any statin at baseline (point of diabetes diagnosis or within the subsequent month) and approximately 1 year thereafter. We defined the latter as receipt of a statin prescription within 12 months after diabetes diagnosis or receipt of a new prescription 12–18 months after diagnosis.

### Sociodemographic and clinical characteristics

We included information on sex, age at type 2 diabetes diagnosis, area-based deprivation, National Health Service (NHS) health board, calendar year of diabetes diagnosis, history of CVD, history of other comorbidities, history of an alcohol use disorder, smoking status, BMI, total cholesterol, SBP and HbA_1c_ at the time of diabetes diagnosis. Sex was determined based on clinical records, thereby capturing a combination of self-reported gender and assigned sex by primary care staff. Area-based deprivation was defined using the Scottish Index of Multiple Deprivation (SIMD) 2020 [23]. The SIMD is derived by dividing Scotland into almost 7000 small geographical areas and using census data on more than 30 indicators across seven domains (income, employment, education, health, access to services, crime and housing) to measure deprivation within each area, after which areas are ranked by deprivation level. We divided the SIMD into deciles for the present study. NHS Scotland comprises 15 regional health boards, but we combined the island health boards for Orkney, Shetland and Western Isles to maintain adequate group size. We used ICD codes and Office of Population Censuses and Surveys Classification (OPCS) codes from acute hospital admission records to identify history of CVD, defined as ischaemic heart disease, cerebrovascular disease or peripheral arterial disease (ESM Table 2) using a 10-year look-back period from diabetes diagnosis. We defined history of other comorbidities (using an adapted Charlson index [24]) and of an alcohol use disorder based on previously developed definitions [10]. We categorised smoking status as current smoker, ex-smoker or non-smoker, based on the record closest to the date of diabetes diagnosis. We ascertained BMI using a window of 6 months before and 3 months after diabetes diagnosis, and ascertained total cholesterol, SBP and HbA_1c_ levels at the time of diabetes diagnosis using a window of 3 months before and after diagnosis, calculating the median when multiple values were recorded.

### Statistical analyses

We summarised baseline characteristics and compared them based on SMI status, using either the χ^2^ test, one-way ANOVA or the Kruskal–Wallis test. For the primary analyses, we used logistic regression to compare the odds of achieving target levels for each of SBP, total cholesterol and HbA_1c_ approximately 1 year after diabetes diagnosis in people with schizophrenia, bipolar disorder or major depression compared to people without a prior hospital admission record for mental illness. We stratified all models by sex, and fitted two models for each cardiovascular risk factor. The first model included sociodemographic factors (age at type 2 diabetes diagnosis, area-based deprivation and NHS health board) and clinical history (calendar year of diagnosis, history of CVD and history of other comorbidities) that were considered potential confounders rather than mediators. In the second model, we additionally included factors that may partially mediate any observed association, including lifestyle-related factors (history of an alcohol use disorder, smoking status and BMI) and total cholesterol, SBP and HbA_1c_ levels at the time of diabetes diagnosis. After visual and statistical exploration of potential interactions, the interaction between age and BMI was included in the second model to improve model fit.

In secondary analyses, we used logistic regression to compare the odds of receiving a statin prescription at the time of type 2 diabetes diagnosis and 1 year thereafter among people with an SMI vs those without each SMI. We initially stratified analyses by sex and history of CVD to assess on sex differences and examine differences between primary and secondary prevention of CVD. However, as the results were similar for men and women and stratification reduced the statistical power, we combined men and women in the final model, with sex included as a covariate. We fitted two models, including the same covariates as described in the primary analysis (with the exception of history of CVD, which we stratified by). For the 1 year analyses, we also included statin prescribing at the time of diabetes diagnosis in the models. All models including lifestyle-related factors included an interaction term for age and BMI.

We used multiple imputation to impute 40 datasets to deal with missing data for six covariates: area-based deprivation, smoking status, BMI, and total cholesterol, SBP and HbA_1c_ levels at baseline (ESM Methods). We analysed each imputed dataset separately and pooled results using Rubin’s rule [25].

### Sensitivity analyses

We repeated primary analyses using the complete-case cohort and compared results to those after multiple imputation. We also repeated primary analyses using the Scottish Intercollegiate Guidelines Network defined target levels for SBP (<130 mmHg) and HbA_1c_ (≤53 mmol/mol, 7%), to assess whether stricter target levels influenced the results [20, 26]. For these analyses, we only used the complete-case cohort.

We performed all analyses using R version 3.6.0 [27]. We used the multiple imputation by chained equations (mice) package 3.14.0 [28] to perform multiple imputation.

### Ethics approval

Permission for the use of pseudonymised data for this research was obtained from a Scottish Multicentre Research Ethics Committee (reference 21/WS/0047) and the Public Benefit and Privacy Panel (reference 1617-0147).

## Results

We included 291,644 people diagnosed with type 2 diabetes, of whom 3024 (1.0%), 1400 (0.5%) and 9721 (3.3%) had a prior hospital admission record for schizophrenia, bipolar disorder and major depression, respectively (Table 1). Overall, the cohort included slightly more men (56.9%) than women, although women were somewhat over-represented in the groups with a history of major depression and bipolar disorder (59.3% and 59.1%, respectively). Compared to people without SMI, people with SMI were younger at the time of diabetes diagnosis and were more likely to live in deprived areas, have a history of an alcohol use disorder and be current smokers. At time of diabetes diagnosis, median BMI and mean total cholesterol were slightly higher, and mean SBP was lower, in people with SMI vs those without SMI. History of CVD and other comorbidities was more common in people with depression and bipolar disorder (but not schizophrenia) than in those without mental illness. HbA_1c_ levels at time of diabetes diagnosis were similar across all groups. Patterns of missing data were largely similar by SMI status.

**Table 1:** Baseline characteristics of adults diagnosed with type 2 diabetes between 2004 and 2020 in Scotland, by SMI status

	No history of a mental disorder (*N*=277,499)	Schizophrenia (*N*=3024)	Bipolar disorder (*N*=1400)	Major depression (*N*=9721)
Female	119,498 (43.1)	1185 (39.2)	827 (59.1)	5766 (59.3)
Age (years)	60.2 ± 13.2	51.9 ± 12.5	56.6 ± 12.3	58.2 ± 12.4
Deprivation^a^				
1 (most deprived)	66,672 (24.0)	1166 (38.6)	426 (30.4)	3342 (34.4)
2	63,480 (22.9)	779 (25.8)	314 (22.4)	2453 (25.2)
3	56,659 (20.4)	558 (18.5)	271 (19.4)	1836 (18.9)
4	49,506 (17.8)	320 (10.6)	233 (16.6)	1208 (12.4)
5 (least deprived)	38,737 (14.0)	183 (6.1)	147 (10.5)	803 (8.3)
Missing	2445 (0.9)	18 (0.6)	9 (0.6)	79 (0.8)
History of CVD	62,391 (22.5)	556 (18.4)	378 (27.0)	3755 (38.6)
Modified Charlson index^b^				
0	236,463 (85.2)	2472 (81.7)	1058 (75.6)	6550 (67.4)
1–8	28,514 (10.3)	415 (13.7)	242 (17.3)	2229 (22.9)
>8	12,522 (4.5)	137 (4.5)	100 (7.1)	942 (9.7)
History of an alcohol use disorder	7994 (2.9)	255 (8.4)	142 (10.1)	1348 (13.9)
Smoking status				
Never smoked	125,381 (45.2)	770 (25.5)	448 (32.0)	3175 (32.7)
Ex-smoker	95,939 (34.6)	665 (22.0)	418 (29.9)	2958 (30.4)
Current smoker	54,572 (19.7)	1558 (51.5)	528 (37.7)	3529 (36.3)
Missing	1607 (0.6)	31 (1.0)	6 (0.4)	59 (0.6)
BMI (kg/m^2^)	31.9 (28.2–36.5)	33.5 (29.5–38.3)	33.5 (29.7–38.4)	33.5 (29.4–38.7)
Missing	57,835 (20.8)	644 (21.3)	304 (21.7)	2145 (22.1)
Total cholesterol (mmol/l)	5.03 ± 1.24	5.37 ± 1.39	5.33 ± 1.39	5.17 ± 1.35
Missing	53,913 (19.4)	635 (21.0)	285 (20.4)	2131 (21.9)
SBP (mmHg)	137 ± 15.7	130 ± 15.2	132 ± 14.9	134 ± 15.7
Missing	36,961 (13.3)	536 (17.7)	235 (16.8)	1430 (14.7)
HbA_1c_				
mmol/mol	56.0 (49.0–74.0)	57.0 (48.5–78.0)	54.0 (48.0–78.0)	55.0 (49.0–72.0)
%	7.3 (6.6–8.9)	7.4 (6.6–9.3)	7.1 (6.5–9.3)	7.2 (6.6–8.7)
Missing	41,743 (15.0)	520 (17.2)	211 (15.1)	1340 (13.8)

Data are means ± SD, *n* (%) or median (IQR)

^a^Deprivation was assessed using the Scottish Index of Multiple Deprivation (SIMD) 2020 [23]

^b^An adapted Charlson index was used to assess history of comorbidities other than CVD [10, 24]

All baseline characteristics differed based on SMI status (*p*<0.001)

### Achievement of risk factor target levels

Across the entire cohort, 53.5%, 57.2% and 57.8% of people met the target levels for total cholesterol (≤5.0 mmol/l), SBP (≤140 mmHg) and HbA_1c_ (<58 mmol/mol, 7.5%), respectively (Table 2). Compared to people without a mental illness, lower proportions of people with each SMI achieved target cholesterol levels. In contrast, a higher proportion of people with SMI achieved target SBP levels.

**Table 2:** Total cholesterol, SBP and HbA_1c_ target level achievement 1 year after type 2 diabetes diagnosis, and statin prescribing at diabetes diagnosis and 1 year thereafter, by SMI status

	No history of a mental disorder (*N*=277,499)	Schizophrenia (*N*=3024)	Bipolar disorder (*N*=1400)	Major depression (*N*=9721)
Total cholesterol ≤5.0 mmol/l	148,488 (53.5)	1464 (48.4)	688 (49.1)	4686 (48.2)
SBP ≤140 mmHg	158,696 (57.2)	1923 (63.6)	883 (63.1)	5954 (61.2)
HbA_1c_ <58 mmol/mol (7.5%)	160,436 (57.8)	1681 (55.6)	853 (60.9)	5465 (56.2)
Statin prescribed at the time of diabetes diagnosis	125,080 (45.1)	1148 (38.0)	597 (42.6)	4778 (49.2)
Statin prescribed 1 year thereafter	172,797 (62.3)	1809 (59.8)	861 (61.5)	6519 (67.1)

Values are *n* (%)

**Fig. 2:**
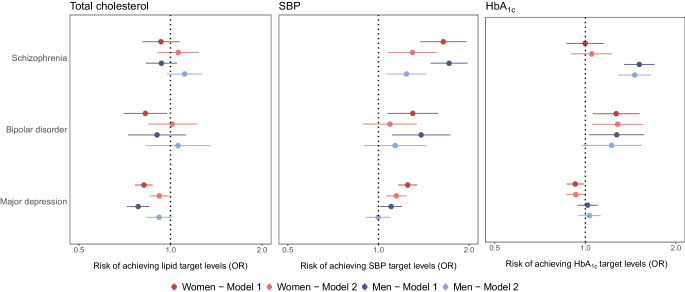
OR for total cholesterol, SBP and HbA_1c_ target level achievement, comparing people with an SMI vs those without each SMI, stratified by sex. Model 1 is adjusted for age at diagnosis, area-based deprivation, NHS health board, calendar year of diagnosis, history of CVD and history of other morbidities; model 2 is additionally adjusted for history of an alcohol use disorder, smoking status, BMI and total cholesterol, SBP and HbA_1c_ at the time of diabetes diagnosis

In the model adjusting for sociodemographic factors and clinical history, the odds of achieving the target cholesterol levels were lower among women with bipolar disorder (OR 0.83; 95% CI 0.70, 0.97) and people with major depression (men: OR 0.78; 95% CI 0.71, 0.85; women: OR 0.82; 95% CI 0.77, 0.87) compared to those without a history of mental illness. There was no statistically significant difference for those with schizophrenia or men with bipolar disorder. Adding lifestyle-related factors to the model attenuated the association between SMI status and achievement of the cholesterol target level (Fig. 2 and ESM Table 3).

Interestingly, people with SMI were more likely to achieve BP target levels than people without mental illness. The effect estimates were statistically significant for all SMI disorders, with the magnitude of effect being greatest for schizophrenia (men: OR 1.72; 95% CI 1.49, 1.98; women: OR 1.64; 95% CI 1.38, 1.96). Again, estimates were attenuated after adding lifestyle-related factors (Fig. 2 and ESM Table 3), but remained statistically significant for men and women with schizophrenia and women with major depression.

Among those with schizophrenia, the odds for HbA_1c_ target level achievement differed by sex. Compared to people without mental illness, men with schizophrenia were more likely to achieve the HbA_1c_ target level (OR 1.51; 95% CI 1.34, 1.70), but there was no difference among women. Both men and women with bipolar disorder had higher odds of achieving the HbA_1c_ target level compared with the comparison groups (men: OR 1.27; 95% CI 1.03, 1.57; women: OR 1.27; 95% CI 1.06, 1.51). In people with major depression, there was no difference in the odds of achieving HbA_1c_ target levels for men but a slightly reduced odds for women (OR 0.93; 95% CI 0.87, 0.99). The effect estimates were very similar after adding lifestyle-related factors to the models (Fig. 2 and ESM Table 3).

### Sensitivity analyses

The results of analyses using the complete-case dataset were very similar to those using the multiple imputed datasets (ESM Table 4). When we applied the stricter target levels defined by the Scottish Intercollegiate Guidelines Network, i.e. SBP <130 mmHg and HbA_1c_ ≤53 mmol/mol (7%), the ORs of achieving target levels were further from the null value (i.e. the differences were greater) than those based on the target levels applied in the Scottish Diabetes Survey (ESM Table 5).

### Statin prescribing

At the time of diabetes diagnosis, 45.1% of the cohort were receiving a statin prescription. This proportion increased to 62.4% 1 year later. The proportion prescribed a statin was highest among people with major depression, both at the time of diabetes diagnosis and 1 year thereafter, followed by people without a history of a mental illness. People with schizophrenia and bipolar disorder were less likely to have received a statin prescription (Table 2).

**Fig. 3:**
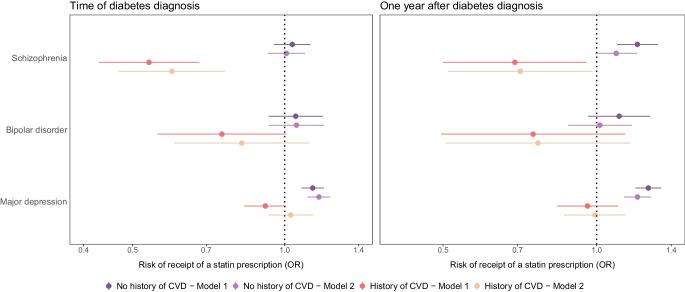
Statin prescribing at the time of diabetes diagnosis and 1 year thereafter, by SMI status. Model adjustment is the same as the models in Fig. 2, with sex included as a covariate and models stratified by history of CVD. The models estimating the association between SMI status and statin prescribing 1 year after diabetes diagnosis also included statin prescribing at the time of diabetes diagnosis as a covariate

Among people without a history of CVD at time of diabetes diagnosis, people with schizophrenia and bipolar disorder had similar odds of having statins prescribed for primary prevention of CVD to those for people without mental illness. Interestingly, people with major depression were more likely to be prescribed statins than those without prior mental illness (OR 1.14; 95% CI 1.08, 1.19). However, among people with a history of CVD, those with SMI were less likely to be prescribed statins compared to people without mental illness (schizophrenia: OR 0.54; 95% CI 0.43, 0.68; bipolar disorder: OR 0.75; 95% CI 0.56, 1.01; major depression: OR 0.92; 95% CI 0.83, 1.01). Further adjustment for additional variables, including lifestyle-related factors, gave similar or attenuated estimates (Fig. 3 and ESM Table 6).

One year after diagnosis of diabetes, among those without a history of CVD, people with SMI were all more likely to receive a statin prescription compared to people without a mental illness. Among those with a history of CVD, the odds of statin prescribing remained lower among people with schizophrenia and bipolar disorder, with no clear difference for those with depression (Fig. 3 and ESM Table 6).

## Discussion

In people with type 2 diabetes, achievement of target levels for cholesterol, BP and HbA_1c_ 1 year after diagnosis of diabetes differed by SMI status, with differing patterns observed across these cardiovascular risk factors and by individual SMI disorder and sex. Compared to people without a mental illness, those with SMI were less likely to achieve target cholesterol levels but more likely to achieve target BP levels. People with bipolar disorder and men with schizophrenia were more likely to achieve the HbA_1c_ target level, but women with major depression were less likely to achieve this target level. The association between SMI status and statin prescribing differed by prior history of CVD. Among people without CVD, those with an SMI were equally or more likely to be prescribed statins compared to those without mental illness, both at diabetes diagnosis and 1 year later. In people with pre-existing CVD, statin prescribing was less likely among people with an SMI vs those without.

Few studies have investigated achievement of lipid target levels by SMI status among people with type 2 diabetes. Two studies found no difference in lipid target level achievement based on SMI status [14, 15], which contrasts somewhat with our findings. However, direct comparison is difficult as the previous studies did not differentiate between SMI diagnoses. Moreover, these studies defined SMI using both primary and secondary care data, and may therefore have included a group with less severe SMI than in our study. If associations differ by severity of SMI, then this may account for these inconsistent findings. Our study confirms variation in associations by individual SMI disorder, and the importance of analysing conditions separately, as previously demonstrated by a Danish cohort study [12]. Interestingly, a fourth study found no difference in lipid target level achievement among those with hospital-diagnosed depression vs no mental illness, whereas people with depression defined based on antidepressant prescription data were more likely to achieve lipid target levels than those without mental illness [17]. In contrast to the results for lipid target achievement, our findings on BP target achievement do align with the results of two previous studies [14, 15]. Additionally, our findings on achievement of HbA_1c_ target levels are somewhat consistent with results of previous studies, which either reported that target level achievement did not differ by mental illness status [14, 15, 17] or that target level achievement was more likely in people with mental illness [12]. Two studies investigated differences by sex, and, in contrast to our findings, did not find any evidence for effect modification by sex [14, 17]. Only one study analysed SMI disorders separately [12] and one included people with depression only [17], with the remainder analysing SMI as a composite group [14, 15].

Our findings on prescription of lipid-modifying drugs (statins) by SMI status align with the few previous studies that have investigated this [16, 17]. A Danish study reported that having a hospital diagnosis of depression was associated with a greater likelihood of initiation of treatment with lipid-modifying drugs, but they did not stratify by history of CVD [17]. Interestingly, a Finnish cohort study [16] found more marked differences in prescription of lipid-modifying drugs by SMI status for secondary prevention of CVD than for primary prevention of CVD [16]. Qualitative studies involving healthcare professionals and patients provide plausible explanations for these findings [29–32]. These explanations include overshadowing of physical health problems and challenges of cardiovascular risk management in people with SMI, especially among patients with more complex issues, such as those with multimorbidity, including CVD [29–32]. Further quantitative and qualitative research is needed to explore the interplay between mental and physical health problems. In particular, studies should investigate how this interplay affects health inequalities in high-risk subgroups such as people with prior CVD or other physical health problems.

It appears paradoxical that people with major depression had the lowest likelihood of achieving total cholesterol target levels whilst being the most likely to have statins prescribed at baseline and 1 year thereafter. This may reflect confounding by indication or a difference in medication adherence, as reported in qualitative studies [30, 31]. Interestingly, Rohde et al used a proxy to assess treatment adherence among people with type 2 diabetes with and without depression, and reported that people with depression appeared to have higher treatment adherence compared to people without a mental illness [17]. This result warrants further investigation to determine whether these findings are replicable in other settings, including Scotland, and to establish whether differences in medication adherence may partly account for our observed findings.

Our study has multiple strengths. We included a representative population-based cohort from the Scottish Diabetes Research Network National Diabetes Dataset, which includes information on >99% of people with diabetes in Scotland. Additionally, our previous research demonstrated that, in Scotland, cardiovascular risk monitoring during the first year after type 2 diabetes diagnosis is similar or better in people with an SMI compared to those without [11], and so we can be confident that differences in cardiovascular risk management are due to differences in the translation of risk monitoring into risk management. Due to the breadth of data included in the Scottish Diabetes Research Network National Diabetes Dataset, we were able to adjust for a wide range of covariates. Finally, the sample size allowed for stratification by sex during primary analyses and history of CVD during secondary analyses, thereby allowing investigation of differences in outcomes within these subgroups, which have rarely been addressed in previous studies. The observed sex differences in achievement of the HbA_1c_ target level are intriguing, and add to the findings of other studies that reported important sex differences in biomedical outcomes among people with diabetes [33].

A limitation of our study is that we defined SMI based on acute and psychiatric hospital records. Therefore, our findings may not be generalisable to people with SMI who have not been admitted to hospital, and whose condition may be less severe. Additionally, we did not account for the development of SMI during the follow-up period. Although unlikely to have affected the findings for schizophrenia and bipolar disorder, which are usually diagnosed at a younger age than type 2 diabetes, this may have affected results for major depression, given the bidirectional association between type 2 diabetes and depression [34]. We also did not have information on other lifestyle-related factors such as physical activity and diet, and so could not explore whether these factors may account for some of the observed associations. We did not adjust for ethnicity due to this information being missing in approximately 9% of patients. Although the general population and subgroup with diabetes in Scotland is predominantly white [35], lack of adjustment for ethnicity may have resulted in residual confounding. Limited data on HDL and LDL levels prohibited analyses of these outcome measures in addition to total cholesterol. We only had information on medication prescribing and not adherence, and so could not determine how statin prescription translates into adherence. It was beyond the scope of the current study to examine the role of psychotropic medication use. Whilst antipsychotic medication and antidepressants are known to have metabolic side-effects, their association with cardiovascular risk factor levels once diabetes has developed is less clear [36]. However, lack of adjustment for psychotropic medication may have led to residual confounding of effect estimates. Finally, almost 40% of individuals had missing data for at least one covariate. We addressed this using multiple imputation. We could not assess target level achievement in approximately 10% of individuals for each risk factor. As using imputed outcome data in analyses is reported to mainly add noise, we excluded these individuals after multiple imputation [37].

Our study makes a valuable contribution to an important but notably under-studied area, and extends our previous work on receipt of diabetes processes of care in Scotland [11]. The findings demonstrate that, in addition to being equally or more likely to receive routine diabetes monitoring (delivered in the primary care setting) [11], people with an SMI are, overall, equally or more likely to achieve BP and HbA_1c_ target levels, but not necessarily cholesterol target levels in the short term following diabetes diagnosis. Our study highlights the importance of investigating these associations by individual SMI disorder and sex, as it reveals novel insights into how the association between SMI and achievement of cardiovascular risk factor targets may vary by these factors. For example, it is interesting that women, but not men, with major depression were slightly less likely to achieve HbA_1c_ target levels within the first year after diabetes diagnosis. As existing data in this area are so limited, further studies need to examine this finding in other populations and settings to confirm or refute these patterns. Moreover, future research should look beyond the short term to examine whether associations between SMI and achievement of cardiovascular risk factor target levels change in the medium and long term, and to explore whether cardiovascular risk management varies between individuals with SMI who do or do not experience subsequent psychiatric admissions during follow-up. This has rarely been investigated, with just one study reporting that HbA_1c_ levels, but not cholesterol levels, increased more in those with an SMI vs those without an SMI in the 1–4 year period after diabetes diagnosis [18]. As also found in a previous study [38], the proportion of all people with type 2 diabetes receiving statins is lower than expected based on Scottish clinical care guidelines [38]. Our findings that statin treatment differences exist among people with SMI and a history of CVD reveal a worrying disparity in this high-risk complex-needs subgroup. This reinforces the importance of clinical review of statin prescribing, particularly for secondary prevention of CVD among people with SMI. Further research to determine the underlying reasons for these disparities is necessary to inform appropriate interventions. In particular, concerns about polypharmacy, which may contribute to the pattern of findings for the high-risk complex-needs subgroup, merit investigation. Future research should also examine, in depth, the role of antipsychotic and antidepressant medication, given their potential for metabolic side-effects, and the role of glucose-lowering treatment, which may vary by SMI status. Additional research steps include investigation of the management of other cardiovascular risk factors, including HbA_1c_ and BP. It is somewhat paradoxical that, in the short term at least, cardiovascular risk factor control is generally similar if not better in people with an SMI vs those without, and yet this vulnerable group have a higher risk of CVD and cardiovascular death [10, 39, 40]. The recommended next research steps will illuminate this complex picture further, particularly the extent to which sub-optimal cardiovascular risk factor management plays a role in SMI disparities in diabetes outcomes.

### Supplementary Information

Below is the link to the electronic supplementary material.

Supplementary file1 (PDF 103 KB)

## Data Availability

Data are available via application to the Scottish Diabetes Research Network Epidemiology Group.

## Abbreviations

NHS: National Health Service (UK)
SBP: Systolic BP
SIMD: Scottish Index of Multiple Deprivation
SMI: Severe mental illness
