# Supplementary material for: Cardiovascular risk management among individuals with type 2 diabetes and severe mental illness: a cohort study
Source: Diabetologia. 2024 Feb 26;67(6):1029–39. doi: 10.1007/s00125-024-06111-w (PMC11058755; doi:10.1007/s00125-024-06111-w)
Supplement: Supplementary file 1 — Supplementary file1 (PDF 103 KB) [file 125_2024_6111_MOESM1_ESM.pdf]

Supplementary material

Cardiovascular risk management among individuals with type 2 diabetes and severe mental illness: a cohort study

Running title: SMI and Cardiovascular risk management

Jonne G. ter Braake[1], Kelly J. Fleetwood[2], Rimke C. Vos[1], Luke Blackbourn[3], Stuart J. McGurnaghan[3], Sarah H. Wild[2], Caroline A. Jackson[2] on behalf of the Scottish Diabetes Research Network Epidemiology Group

[1]Department of Public Health and Primary Care, Leiden University Medical Centre, The Hague, The Netherlands

[2]Usher Institute, University of Edinburgh, Edinburgh, UK

[3]MRC Institute of Genetics and Cancer, University of Edinburgh, Edinburgh, UK.

Electronic supplementary material

ESM Table 1: Hierarchy of mental health conditions, including the ICD-9 and ICD-10 codes to identify them from hospital admission records

Position in hierarchy	Mental health condition	ICD-9 codes	ICD-10 codes
1	Schizophrenia	295.0–295.3, 295.5–295.9	F20, F25
2	Bipolar disorder	296.0, 296.2–296.6	F30–F31
3	Major depression*	296.1, 298.0, 300.4, 311	F32–F33
Excluded from analyses	Other psychoses: schizotypal disorders, acute and transient psychosis, delusional disorders, and other psychotic disorders<br><br>Other mental health conditions: including other mood disorders, neuroses, dissociative disorders, somatoform disorders, eating disorders, non–organic sleep disorders and other behavioural syndromes associated with physiological disturbances and physical factors, disorders of adult personality and behaviour, disorders of psychological development, behavioural and emotional disorders with onset in childhood and adolescence and unspecified mental disorders	295.4, 297.0–297.9, 298.3, 298.4, 298.8, 298.9<br>293.8, 296.9, 298.1, 298.2, 299.0–300.3, 300.5–301.9, 302.1–302.9, 306.0–309.9, 312.0–315.9, 316	F21–F24, F28, F29<br><br>F34–F69, F80–F99

* The International Classification of Diseases 9th Revision (ICD-9) and -10 codes used to include major depression are not all specific for major depression, but can refer to depression in general. Since acute and psychiatric hospital records are used to determine the mental disorder, it can be assumed that the vast majority of people had major depression.

This hierarchy and the definitions of the mental health conditions were adapted from Fleetwood et al. [1].

ESM Table 2: ICD-9, ICD-10, and OPCS-4 codes used to define the history of CVD

Description	ICD-9 code	ICD-10 code	OPCS-4
Ischaemic heart disease	410–414	I20–I25	K40–K46, K49, K50, K63.1–K63.3, K65, K75
Cerebrovascular disease	430, 431, 433–435, 438	I60, I61, I63–I66, I69 G45	L29.4, L29.5, L31.4

			ICD–10 I63 AND OPSC-4 X83.3 OR L35.4
Peripheral arterial disease	440, 443, 444, 447	I70, I73, I74, I77	L50, L51, L52, L53.0–L53.2, L54.1, L54.2, L54.4, L54.8, L54.9, L58, L59, L60, L62.1, L62.2, L62.8, L62.9, L63.1–L63.3, L63.5, L63.8, L63.9, L65

The definitions are based on previous literature [1–3].

ESM Methods: The multiple imputation process and rationale

We used a parallel version of the multiple imputation by chained equations (MICE) package 3.14.0 to perform multiple imputation. Data were missing for area-based deprivation, systolic blood pressure at baseline, total cholesterol levels at baseline, HbA_{1c} levels at baseline, BMI, and smoking status. It was assumed that data were missing at random (MAR).

As there seemed to be an interaction between age and sex and between age and BMI, these interaction terms were included during multiple imputation as well [4, 5]. To deal with the interaction between age and sex, the dataset was split based on sex, whereafter multiple imputation was run on both subsets and the products were bound together. For the interaction between age at type 2 diabetes diagnosis and BMI, the interaction term was included as an additional variable by subtracting the mean and taking the product of age and BMI [5]. When BMI was missing, and thus the interaction term was missing, the interaction would be calculated from the imputed value of BMI, instead of predicted based on the other available data, to prevent convergence as a result of a feedback loop between BMI and the interaction term.

Both the continuous and dichotomised outcomes were included in the multiple imputation process [6]. When the outcome was missing, the predicted continuous outcome was not used in the prediction process of the dichotomised outcome, and vice versa, to prevent convergence. Available data from individuals that miss outcomes were used during the multiple imputation process, whereafter four sets of the imputed datasets were formed, one for each primary outcome and one dataset with all individuals for the secondary outcome, statin prescription, since there was no missing outcome data for this outcome [4, 7].

As a rule of thumb, the number of generated datasets should be at least equal to the percentage of incomplete cases, as 38.3% of participants had at least one of the above-mentioned covariates missing, 40 datasets were imputed [5, 7]. We analysed each imputed dataset separately and pooled results using Rubin's rule [8].

ESM Table 3: Odds ratios (95% CI) for total cholesterol, systolic blood pressure, and HbA_{1c} target level achievement, comparing people with each severe mental illness versus no mental illness, stratified by sex.

Outcome	Model	Schizophrenia	Bipolar disorder	Major depression
**Lipid target level achievement** (total cholesterol ≤ 5.0 mmol/l)	Model 1 – Male	0.92 (0.83–1.05)	0.90 (0.73–1.12)	0.78 (0.71–0.85)
	Model 1 – Female	0.93 (0.81–1.07)	0.83 (0.70–0.97)	0.82 (0.77–0.87)
	Model 2 – Male	1.11 (0.97–1.27)	1.06 (0.83–1.35)	0.92 (0.83–1.01)
	Model 2 – Female	1.06 (0.91–1.24)	1.01 (0.84–1.22)	0.92 (0.85–0.99)
**Blood pressure target level achievement** (systolic blood pressure ≤ 140 mmHg)	Model 1 – Male	1.72 (1.49–1.98)	1.39 (1.11–1.73)	1.10 (1.01–1.20)
	Model 1 – Female	1.64 (1.38–1.96)	1.30 (1.07–1.58)	1.25 (1.16–1.34)
	Model 2 – Male	1.23 (1.06–1.44)	1.14 (0.90–1.44)	1.00 (0.91–1.09)
	Model 2 – Female	1.30 (1.07–1.56)	1.09 (0.89–1.34)	1.15 (1.06–1.24)
**Glucose target level achievement** (HbA_{1c} < 58 mmol/mol (7.5%))	Model 1 – Male	1.51 (1.34–1.70)	1.27 (1.03–1.57)	1.02 (0.94–1.10)
	Model 1 – Female	1.00 (0.87–1.15)	1.27 (1.06–1.51)	0.93 (0.87–0.99)
	Model 2 – Male	1.46 (1.29–1.65)	1.22 (0.98–1.53)	1.03 (0.95–1.12)
	Model 2 – Female	1.05 (0.90–1.23)	1.28 (1.05–1.55)	0.93 (0.87–1.00)

Model 1 is adjusted for age at diagnosis, area-based deprivation, NHS health board, calendar year of diagnosis, history of CVD, and history of other morbidities. Model 2 is additionally adjusted for history of an alcohol use disorder, smoking status, body mass index, and total cholesterol, systolic blood pressure, and HbA_{1c} at time of diabetes diagnosis. CI = confidence interval; OR = odds ratio

ESM Table 4: Odds ratios (95% CI) from the sensitivity analysis including the complete-case cohort for total cholesterol, systolic blood pressure, and HbA_{1c} target level achievement comparing people with each severe mental illness versus no mental illness, stratified by sex.

Outcome	Model	Schizophrenia	Bipolar disorder	Major depression
**Lipid target level achievement** (total cholesterol ≤5.0 mmol/l)	Model 1 – Male	0.94 (0.84–1.06)	0.90 (0.72–1.12)	0.78 (0.72–0.85)
	Model 1 – Female	0.92 (0.80–1.06)	0.82 (0.70–0.97)	0.81 (0.76–1.06)
	Model 2 – Male	1.27 (1.08–1.50)	1.30 (0.95–1.81)	0.95 (0.84–1.07)
	Model 2 – Female	1.17 (0.97–1.42)	0.98 (0.71–1.12)	0.92 (0.84–1.01)
**Blood pressure target level achievement** (systolic blood pressure ≤140 mmHg)	Model 1 – Male	1.73 (1.51–2.00)	1.39 (1.11–1.74)	1.11 (1.02–1.20)
	Model 1 – Female	1.66 (1.39–1.98)	1.33 (1.10–1.61)	1.25 (1.17–1.35)
	Model 2 – Male	1.30 (1.08–1.58)	1.34 (0.98–1.86)	1.02 (0.91–1.15)
	Model 2 – Female	1.23 (0.98–1.57)	1.09 (0.84–1.42)	1.09 (0.99–1.21)
	Model 1 – Male	1.51 (1.34–1.70)	1.29 (1.05–1.59)	1.01 (0.94–1.10)

**Glucose target level achievement** (HbA_{1c} <58 mmol/mol (7.5%))	Model 1 – Female	1.00 (0.86–1.15)	1.26 (1.06–1.51)	0.91 (0.86–0.98)
	Model 2 – Male	1.43 (1.23–1.68)	1.38 (1.03–1.88)	1.05 (0.95–1.17)
	Model 2 – Female	1.03 (0.85–1.26)	1.34 (1.04–1.74)	0.90 (0.83–0.99)

Model 1 is adjusted for age at diagnosis, area–based deprivation, NHS health board, calendar year of diagnosis, history of CVD, and history of other morbidities. Model 2 is additionally adjusted for history of an alcohol use disorder, smoking status, body mass index, and total cholesterol, systolic blood pressure, and HbA_{1c} at time of diabetes diagnosis. CI = confidence interval; OR = odds ratio

ESM Table 5: Odds ratios (95% CI) from the sensitivity analysis including SIGN defined target level achievement.

Outcome	Model	Schizophrenia	Bipolar disorder	Major depression
**Blood pressure target level achievement** (systolic blood pressure <130 mmHg)	Model 1 – Male	1.80 (1.61–2.00)	1.50 (1.24–1.80)	1.20 (1.12–1.29)
	Model 1 – Female	1.95 (1.71–2.23)	1.50 (1.28–1.76)	1.28 (1.20–1.36)
	Model 2 – Male	1.31 (1.13–1.51)	1.52 (1.17–1.96)	1.05 (0.95–1.16)
	Model 2 – Female	1.57 (1.31–1.89)	1.26 (1.02–1.57)	1.12 (1.01–1.22)
**Glucose target level achievement** (HbA_{1c} <53 mmol/mol (7%))	Model 1 – Male	1.67 (1.50–1.86)	1.42 (1.17–1.73)	1.06 (0.99–1.14)
	Model 1 – Female	1.16 (1.02–1.33)	1.46 (1.24–1.72)	1.01 (0.95–1.07)
	Model 2 – Male	1.66 (1.43–1.92)	1.46 (1.12–1.92)	1.06 (0.97–1.17)
	Model 2 – Female	1.12 (0.94–1.35)	1.56 (1.24–1.97)	1.01 (0.93–1.10)

Model 1 is adjusted for age at diagnosis, area-based deprivation, NHS health board, calendar year of diagnosis, history of CVD, and history of other morbidities. Model 2 is additionally adjusted for history of an alcohol use disorder, smoking status, body mass index, and total cholesterol, systolic blood pressure, and HbA_{1c} at time of diabetes diagnosis. CI = confidence interval; OR = odds ratio

ESM Table 6: Odds ratios (95% CI) for receipt of a statin prescription at the time of diabetes diagnosis and one year thereafter, comparing people with each severe mental illness versus no mental illness, stratified by history of cardiovascular disease.

Outcome	Model	Schizophrenia	Bipolar disorder	Major depression
**Statin prescribing at the time of diabetes diagnosis**	Model 1 – No history of CVD	1.04 (0.95–1.12)	1.05 (0.93–1.19)	1.14 (1.08–1.19)
	Model 1 – History of CVD	0.54 (0.43–0.68)	0.75 (0.56–1.01)	0.92 (0.83–1.01)
	Model 2 – No history of CVD	1.01 (0.93–1.10)	1.06 (0.93–1.20)	1.17 (1.11–1.23)
	Model 2 –History of CVD	0.60 (0.47–0.76)	0.82 (0.60–1.12)	1.03 (0.93–1.14)
**Statin prescribing one year thereafter**	Model 1 – No history of CVD	1.20 (1.10–1.32)	1.11 (0.96–1.27)	1.26 (1.19–1.34)
	Model 1 – History of CVD	0.69 (0.50–0.96)	0.75 (0.50–1.14)	0.96 (0.84–1.10)

	Model 2 – No history of CVD	1.09 (1.00–1.20)	1.01 (0.88–1.17)	1.20 (1.13–1.28)
	Model 2 –History of CVD	0.71 (0.51–0.98)	0.77 (0.51–1.16)	0.99 (0.86–1.14)

Model 1 is adjusted for age at diagnosis, area-based deprivation, NHS health board, calendar year of diagnosis, history of CVD, and history of other morbidities. Model 2 is additionally adjusted for history of an alcohol use disorder, smoking status, body mass index, and total cholesterol, systolic blood pressure, and HbA_{1c} at the time of diabetes diagnosis. The models estimating the association between SMI status and statin prescribing one year after diabetes diagnosis also included statin prescribing at the time of diabetes diagnosis as a covariate.

References

1. Fleetwood KJ, Wild SH, Licence KAM, Mercer SW, Smith DJ, Jackson CA (2023) Severe Mental Illness and Type 2 Diabetes Outcomes and Complications: A Nationwide Cohort Study. Diabetes Care 46(7):1363–1371. https://doi.org/10.2337/dc23-0177

2. Wright FL, Cheema K, Goldacre R, et al (2022) Effects of the COVID-19 pandemic on secondary care for cardiovascular disease in the UK: an electronic health record analysis across three countries. Eur Heart J Qual Care Clin Outcomes. https://doi.org/10.1093/ehjqcco/qcac077

3. Kuan V, Denaxas S, Gonzalez-Izquierdo A, et al (2019) A chronological map of 308 physical and mental health conditions from 4 million individuals in the English National Health Service. Lancet Digit Health 1(2):e63–e77. https://doi.org/10.1016/S2589-7500(19)30012-3

4. Austin PC, White IR, Lee DS, van Buuren S (2021) Missing Data in Clinical Research: A Tutorial on Multiple Imputation. Canadian Journal of Cardiology 37:1322–1331

5. Buuren S van (2018) Flexible Imputation of Missing Data. Second edition. https://stefvanbuuren.name/fimd/. Accessed 12 Apr 2023

6. Grobler AC, Lee K (2020) Multiple imputation in the presence of an incomplete binary variable created from an underlying continuous variable. Biometrical Journal 62(2):467–478. https://doi.org/10.1002/bimj.201900011

7. White IR, Royston P, Wood AM (2011) Multiple imputation using chained equations: issues and guidance for practice. Stat Med 30(4):377–399. https://doi.org/10.1002/sim.4067

8. Rubin D (1987) Multiple imputation for nonresponse in surveys. New York: Wiley. https://doi.org/10.1002/9780470316696.fmatter
